# Non-additive modulation of synaptic transmission by serotonin, adenosine, and cholinergic modulators in the sensory thalamus

**DOI:** 10.3389/fncel.2015.00060

**Published:** 2015-03-16

**Authors:** Ya-Chin Yang, Chun-Chang Hu, Yi-Chen Lai

**Affiliations:** ^1^Department of Biomedical Sciences, College of Medicine, Chang Gung UniversityTao-Yuan, Taiwan; ^2^Graduate Institute of Biomedical Sciences, College of Medicine, Chang Gung UniversityTao-Yuan, Taiwan; ^3^Department of Neurosurgery, Chang-Gung Memorial HospitalLinkou, Taiwan

**Keywords:** neuromodulation, sensory responsiveness, short-term synaptic plasticity, sleep-wakefulness regulation, thalamocortical network

## Abstract

The thalamus relays sensory information to the cortex. Oscillatory activities of the thalamocortical network are modulated by monoamines, acetylcholine, and adenosine, and could be the key features characteristic of different vigilance states. Although the thalamus is almost always subject to the actions of more than just one neuromodulators, reports on the modulatory effect of coexisting neuromodulators on thalamic synaptic transmission are unexpectedly scarce. We found that, if present alone, monoamine or adenosine decreases retinothalamic synaptic strength and short-term depression, whereas cholinergic modulators generally enhance postsynaptic response to presynaptic activity. However, coexistence of different modulators tends to produce non-additive effect, not predictable based on the action of individual modulators. Acetylcholine, acting via nicotinic receptors, can interact with either serotonin or adenosine to abolish most short-term synaptic depression. Moreover, the coexistence of adenosine and monoamine, with or without acetylcholine, results in robustly decreased synaptic strength and transforms short-term synaptic depression to facilitation. These findings are consistent with a view that acetylcholine is essential for an “enriched” sensory flow through the thalamus, and the flow is trimmed down by concomitant monoamine or adenosine (presumably for the wakefulness and rapid-eye movement, or REM, sleep states, respectively). In contrast, concomitant adenosine and monoamine would lead to a markedly “deprived” (and high-pass filtered) sensory flow, and thus the dramatic decrease of monoamine may constitute the basic demarcation between non-REM and REM sleep. The collective actions of different neuromodulators on thalamic synaptic transmission thus could be indispensable for the understanding of network responsiveness in different vigilance states.

## Introduction

The thalamus relays sensory information to the cortex. The thalamocortical oscillatory activity shows circadian as well as ultradian rhythms and has long been assumed to underlie the switch between different vigilance states (Steriade et al., 1993; Timofeev and Bazhenov, 2005; Llinas and Steriade, 2006; Magnin et al., 2010; Gemignani et al., 2012; Poulet et al., 2012; David et al., 2013). The thalamocortical network activity is modulated by chemicals from both distant and local sources. It has been widely accepted that the potential sleep-wakefulness regulatory neuromodulators such as monoamines, acetylcholine, and adenosine would alter the membrane potential of thalamic relay neurons and may consequently result in switches of firing patterns linked to different sleep-wakefulness states (Coulon et al., 2012). For instance, aminergic and cholinergic neuromodulators were found to depolarize thalamocortical relay neurons, leading to a transition from burst to tonic mode of discharges (McCormick and Bal, 1997; Steriade et al., 1997; Destexhe and Sejnowski, 2003) and presumably more faithful thalamic relay of sensory information to the cerebral cortex. Conversely, adenosine, a presumable somnogen, hyperpolarizes neurons in the sensory thalamus (as in many other brain areas), consistent with the finding that the thalamic neurons tend to fire in the burst mode during the (slow-wave) sleep state (Pape, 1992; Scanziani et al., 1992; Pan et al., 1994, 1995; Wu and Saggau, 1994; Zhang and Schmidt, 1999).

In addition to intrinsic membrane properties, neuromodulators may also have a strong impact on synaptic transmission and thus postsynaptic neuronal activities. Monoamines such as serotonin and dopamine could characteristically decrease synaptic inputs to thalamic relay neurons (Seeburg et al., 2004; Govindaiah and Cox, 2005, 2006). Surprisingly, somnogen adenosine also decreases retinothalamic synaptic transmission in a way very similar to the wake-promoting monoamines (Yang et al., 2014). In this regard, it is interesting that co-activation of presynaptic A1 and 5-HT1 receptors would produce a dramatic synergistic inhibitory effect on thalamic synaptic transmission that is not achievable by either modulator alone but is well suitable for a hypnagogic condition (Yang et al., 2014). These intriguing findings suggest the inadequacy of investigations based on the presence of only one single modulator, and underscore the importance to study the effect of different combinations of two or more modulators simulating different physiological conditions. In any case, the thalamus is almost always subject to the actions of two or more rather than just one neuromodulators, as the basic vigilance states in the sleep-wakefulness cycle are all characterized by different combinations of predominant neuromodulators in the thalamus. Both monoamines and acetylcholine are the major neuromodulators in the thalamus during wakefulness (Jouvet, 1969; Strecker et al., 2000; Lucas-Meunier et al., 2003; Villablanca, 2004; Saper et al., 2005; Haas and Lin, 2011; Berridge et al., 2012). In contrast, adenosine could be the hallmark neuromodulator of sleep (Benington and Heller, 1995; Dobolyi et al., 2000; Porkka-Heiskanen et al., 2000; Krueger et al., 2008; Elmenhorst et al., 2009; Halassa, 2011), with the presence or absence of concomitant monoamines playing a decisive role in the differentiation between rapid-eye movement (REM) sleep and non-REM (NREM) sleep (Hobson et al., 1975; McCarley and Hobson, 1975; Kubin, 2001; Sakai et al., 2001; Pace-Schott and Hobson, 2002).

Although different combinations of monoamine, acetylcholine, and adenosine may play a major role in vigilance state-dependent changes in thalamocortical activities (McCormick, 1992; Benington and Heller, 1995; Porkka-Heiskanen et al., 2000), reports on the modulatory effect of coexisting neuromodulators on the synaptic transmission in the thalamus are unexpectedly scarce. In this study, we demonstrate non-additive effects of monoamines, acetylcholine, and adenosine. Acetylcholine acting through nicotinic receptors can interact with either serotonin or adenosine to abolish most short-term synaptic depression. The coexistence of adenosine, monoamine, and acetylcholine results in robustly decreased synaptic strength and transforms short-term synaptic depression to facilitation, ensuring limited flow and high-pass filtering of sensory information through the thalamus.

## Materials and methods

### Brain slice preparation

All experiments involving animals were carried out in accordance with NIH guidelines and approved by the Committee on the Ethics of Animal Experiments of Chang Gung University, Taiwan. All surgery was performed under isoflurane anesthesia, and all efforts were made to minimize suffering and to reduce the number of animals used. The brain slice containing both the dorsal lateral geniculate nucleus (dLGN) of thalamus and the optic tract was freshly prepared with the procedures described previously (Turner and Salt, 1998; Chen and Regehr, 2000; Yang et al., 2014). Briefly, the whole brain was quickly removed from the C57/Bl6 mouse of either sex (aged postnatal days of 21–30, BioLASCO Taiwan Co., Ltd, Taipei, Taiwan) and immersed into a ice-cold oxygenated cutting solution (in mM, containing 87 NaCl, 37.5 choline chloride, 25 NaHCO3, 25 glucose, 2.5 KCl, 1.25 NaH2PO4, 7 MgCl2, and 0.5 CaCl2). 250–270 μm thick parasagittal slices were obtained using a vibratome (Leica VT1200S; Leica, Nussloch, Germany) and a sapphire blade (DDK). The slices were incubated in the oxygenated cutting solution for 25 min at 30°C and then transferred into an oxygenated saline (in mM, containing 125 NaCl, 26 NaHCO3, 25 glucose, 2.5 KCl, 1.25 NaH2PO4, 1 MgCl2, and 2 CaCl2) for 25 min at 30°C before electrophysiological recordings.

### Electrophysiological recording

Whole-cell voltage-clamp recordings from dLGN thalamocortical neurons were performed using glass pipettes of 1.0–1.5 MΩ filled with an internal solution consisting of (in mM): 35 CsF, 100 CsCl, 10 EGTA, 10 HEPES, 0.1 D600 (methoxyverapamil hydrochloride; Sigma, St. Louis, MO), pH 7.4. The recordings were done following the methods described previously (Chen and Regehr, 2000; Yang et al., 2014) at room temperature (24 ± 1°C) in isolation of corticothalamic circuitry by a razor incision. Current-clamp recordings were obtained at a bath temperature of 34–36°C using glass pipettes (~2.0 MΩ) containing the following internal solution (in mM): 116 KMeSO4, 6 KCl, 2 NaCl, 20 HEPES, 0.5 EGTA, 4 MgATP, 0.3 NaGTP, 10 NaPO4 creatine, and pH 7.25 adjusted with KOH. For study of the retinothalamic glutamatergic transmission, the bath saline solution contained the GABA_A_ receptor antagonist bicuculline (20 μM; Sigma, St. Louis, MO) and, in experiments involving trains, also the GABA_B_ receptor antagonist (2S)-3[[(1S)-1-(3,4-dichlorophenyl)ethyl]amino-2-hydroxypropyl](phenylmethyl) phosphinic acid (CGP55845; Tocris Cookson, Ballwin, MO). The optic tract was stimulated using a pair of glass electrodes filled with saline. The retinogeniculate synapse showed discrete, step-like incremental increases in the postsynaptic currents with gradually increased intensities of the optic tract stimulation (Chen and Regehr, 2000). Therefore, it is possible to obtain the postsynaptic response to the stimulation of a single or a small number of presynaptic fiber(s). Stimulus intensities (10–40 μA, 0.2–0.3 ms) were adjusted until the minimal stimulation that elicited a synaptic response (i.e., the “single-fiber” stimulation) was found, and only the responses to single-fiber stimuli were included for this study (Chen and Regehr, 2000). If not particularly noted in the text, the standard stimulation protocol was 50 ms-separated pairs of stimulation applied by a low frequency of 0.04 Hz to ensure complete synaptic recovery between stimulations. Recordings were acquired with Multiclamp 700B amplifier (MDS Analytical Technologies) filtered at 5 kHz and digitized at 10–20 kHz with a Digidata-1440 analog/digital interface (MDS Analytical Technologies). Data analysis was performed using pCLAMP software (MDS Analytical Technologies). Leak currents, membrane capacitance and access resistance were continuously monitored to ensure constancy throughout every experiment. The experiments with the fluctuation of access resistance over 2 MΩ were rejected. Moreover, the experiments with continuously increasing access resistance during the time of drug application were also not included. Pharmacological agents were purchased from Tocris or Sigma and were dissolved in DMSO or distilled water to make stock solutions, which were stored at −20°C, and diluted 1:1000–1:3000 into the bath reservoir immediately before application. Constant bath flow was ensured via a perfusion pump (Gilson Medical Electric, Middleton, WI) at stable flow rates of 5 ml/min.

### The choice of the modulators and their concentrations

**Figure 1:**
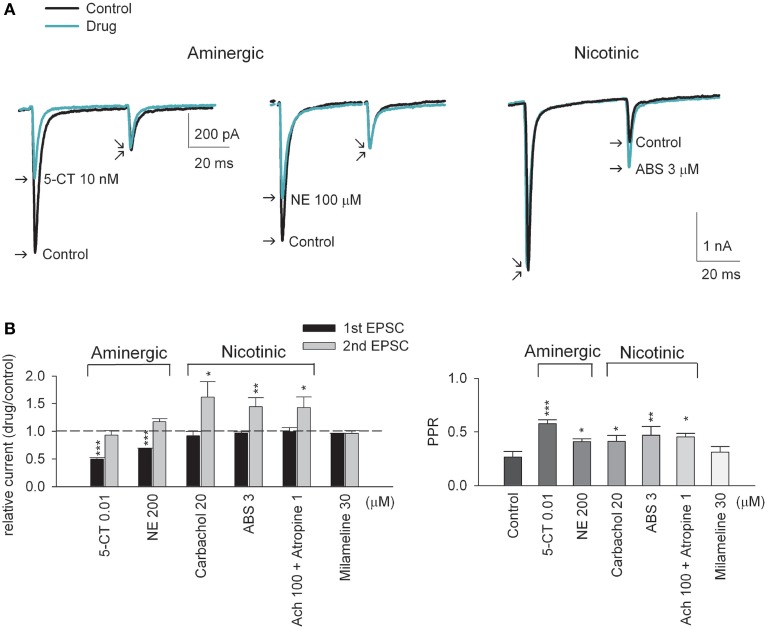
**Aminegic and nicotinic activation alters paired-pulse excitatory postsynaptic currents (EPSCs) differently. (A)** Representative AMPAR current traces recorded at −70 mV from the same neuron in response to presynaptic paired-pulse stimulation in the absence (control, black lines) or presence of a neuromodulator (green lines). The interstimulus interval is 50 ms. Aminergic 5-CT (10 nM, left panel) and NE (100 μM, middle panel, the same scale bars as that in the left panel) decreases the first pulse-evoked EPSC amplitude without significantly affecting the second pulse-evoked EPSC. In contrast, nicotinic ABS (3 μM, right panel) selectively increases the second EPSC amplitude but not the first EPSC. Each trace is the average of three consecutive trials. Stimulus artifacts are omitted for clarity. **(B)** The effects of different neuromodulators on paired-pulse AMPAR currents are compared (*n* = 5–6). Relative current is measured as the peak of 1st and 2nd EPSC amplitude in drug relative to that in control, respectively (left panel). The dashed horizontal line indicates the level where relative EPSC equals 1. PPR (paired-pulse ratio) is measured as the ratio of the peak amplitude of 2nd EPSC to that of 1st EPSC (right panel). ^*^p < 0.05, ^**^p < 0.01, ^***^p < 0.001 compared to control (without drug) by Student's paired *t*-test. See also Figure S1 for the data from individual experiments.

**Figure 2:**
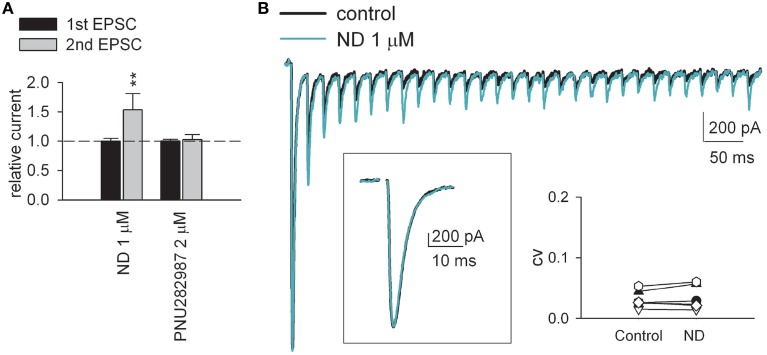
**α4β2 nicotinic acetylcholine receptor agonists but not α7 receptor agonists selectively increase EPSC evoked by the subsequent rather than the first presynaptic stimuli**. **(A)** α4β2 nAchR-selective agonist (−)-nicotine ditartrate (ND) individually shows synaptic effects very much analogous to those of ABS. ND (1 μM) dramatically enhances the second EPSC but not the first EPSC (*n* = 5). On the other hand, highly-selective α7 nAchR agonist PNU 282987 (2 μM, *n* = 5) lacks such effects (on both synaptic currents and PPR). ^**^p < 0.01 compared to control (without drug) by Student's paired *t*-test. See also Figure S1 for the data from individual experiments. **(B)** AMPAR current traces from a representative dLGN relay neuron in the absence (control, black line) or presence of 1 μM ND (green line) in response to a 50 Hz-train of 30-stimuli. Stimulus artifacts are omitted for clarity. ND (1 μM) selectively increases the second and later EPSCs upon a train of continuous stimuli without discernable change in the basic synaptic strength (see *box*: The enlarged first EPSCs evoked by the first stimulus of the 50 Hz train remain the same in control and in 1 μM ND). *Inset*, Consistent with unchanged initial probability of release, we found no significant alteration of the coefficient of variation (CV) of AMPAR EPSC before and after the application of ND. CV is calculated as SD/mean obtained by averaging 12 consecutive EPSCs. CVs from 7 cells in different experiments show no significant changes before and after the application of ND (*p* = 0.798, One-Way ANOVA).

Cholinergic and serotoninergic innervations and the expression of adenosine A1, serotonin 5-HT1, and α4β2 nicotinic acetylcholine receptors have been demonstrated in dLGN (De Lima and Singer, 1987; Fastbom et al., 1987a,b; Papadopoulos and Parnavelas, 1990; Sijbesma et al., 1991; Dinopoulos et al., 1995; Fredholm, 1995; Varnäs et al., 2005; Parent and Descarries, 2008; Hillmer et al., 2013; Yang et al., 2014). Natural neuromodulators were investigated first, and then the specific receptors for the natural neuromodulators were indentified to more specifically characterize the combined effects of different modulators (also see Figure 1B, Yang et al., 2014). The agonists and their concentrations were carefully selected based on the results of characterizations of the concentration-dependent effects of modulators and the corresponding receptor subtypes in our system (Chen and Regehr, 2003; Seeburg et al., 2004; Yang et al., 2014). Detailed pharmacological studies have demonstrated that adenosine and serotonin act through presynaptic A1 and 5-HT1 receptors (but not other subtypes of receptors) at the retinogeniculate synapse, respectively (Chen and Regehr, 2003; Seeburg et al., 2004; Yang et al., 2014). Therefore, the A1 and 5-HT1 receptor agonists were directly used in this study. Receptor subtypes responsible for the cholinergic effect on this synapse were characterized in this study (Figures 1, 2). Because of the technical difficulty in obtaining the exact local concentration of a neuromodulator surrounding a synapse (even with microdialysis), saturating concentrations of each drug was chosen to ensure full activation of specific receptors to demonstrate the non-linear synergistic actions of combined modulators (refer to Yang et al., 2014 for details).

### Statistical analysis

All statistics are provided as mean ± standard error of mean. Numerical data and statistical analyses were managed with pCLAMP (MDS Analytical Technologies), SigmaPlot (Systat Software Inc.), and Excel (Microsoft) programs. Detailed statistical methods are described in figure legends. For all comparisons, *p* < 0.05 was accepted as indicating significant differences.

## Results

### Aminergic and cholinergic modulators show distinct effects on retinothalamic synaptic transmission

**Figure 3:**
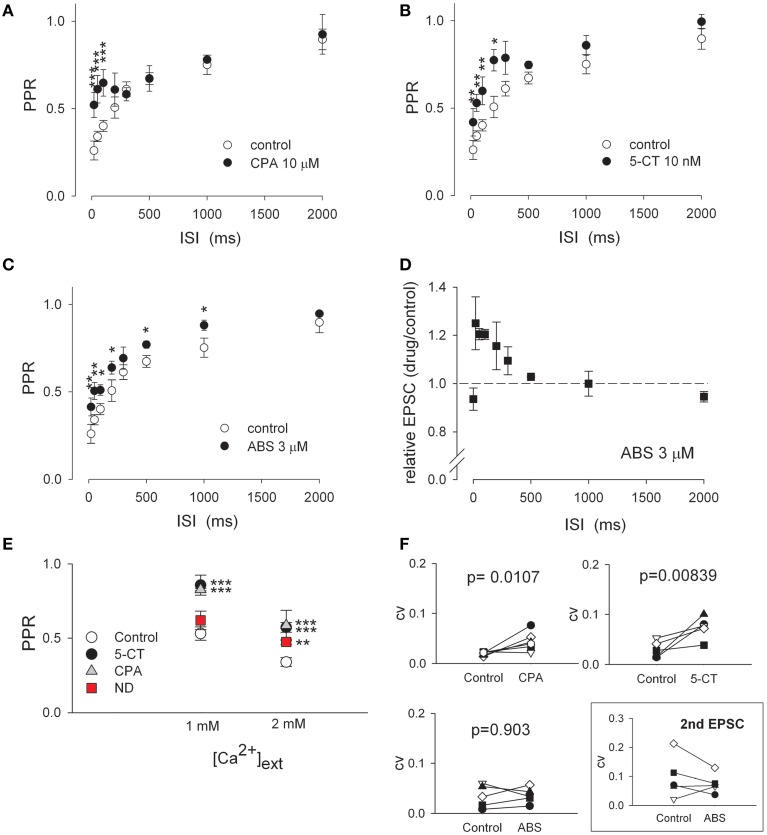
**The recovery time course and Ca^2+^ dependence of short-term depression indicate the similarities between adenosine and aminergic modulation and dissimilarities between aminergic and cholinergic modulation. (A–C)** The stimulus interval-dependent short-term synaptic depression is differently modulated by different neuromodulators. Pairs of AMPAR EPSCs are recorded at varying ISIs in the absence (control, *n* = 10) and presence of 10 μM CPA (part A, *n* = 7), 10 nM 5-CT (part B, *n* = 5), or 3 μM ABS (part C, *n* = 5). PPRs are then plotted against ISI. Saturating concentrations of these drugs are used. ^*^p < 0.05, ^**^p < 0.01, ^***^p < 0.001 compared to control (without drug) by unpaired Student *t*-test. **(D)** The peak amplitude of the second AMPAR EPSC in 3 μM ABS relative to that in control is plotted against ISI. The data at ISI of 0 represents the peak current ratio of the first EPSC in ABS relative to that in control. The dashed horizontal line indicates the level where relative EPSC equals 1. **(E)** α4β2 nAchR agonist (−)-nicotine ditartrate (ND) shows synaptic effects in a Ca^2+^-dependent manner. With 2 mM of extracellular Ca^2+^, 1 μM ND, 10 nM 5-CT, and 10 μM CPA all show significant effects on PPR of AMPAR currents (data are from Figures 1B, 2A, 4B). Lowering extracellular Ca^2+^ concentration (from 2 to 1 mM) increases PPR in control but abolishes the relative effect of 1 μM ND (*n* = 5) on PPR to control. In contrast, lower Ca^2+^ concentration does not significantly alter the relative effect of 10 nM 5-CT or 10 μM CPA to control (*n* = 5 for each drug). For simplicity, all PPR-values obtained in control condition were averaged to give the control data point in experiments with either 1 or 2 mM extracellular Ca^2+^. The interstimulus interval is 50 ms. ^**^p < 0.01, ^***^p < 0.001 for each drug compared to its own control by paired Student *t*-test. **(F)** The coefficient of variation (CV) of AMPAR EPSC before and after the application of 10 μM CPA (*n* = 6), 10 nM 5-CT (*n* = 5), or 3 μM ABS (*n* = 5). CV is calculated as SD/mean obtained by averaging 10 consecutive EPSCs. *P*-values shown in the plots are obtained with One-Way ANOVA. The box shows the CV of 2nd AMPAR EPSC upon paired-pulse stimulation. There is a vague tendency of decreased CV by 3 μM ABS (*n* = 5), although a statistical significance is not reached (*P* = 0.572).

**Figure 4:**
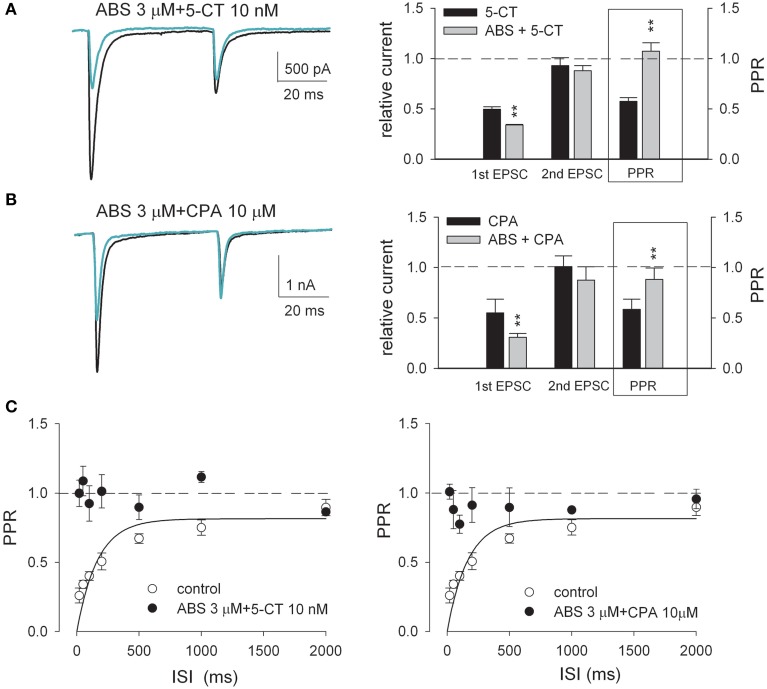
**The paired-pulse synaptic plasticity is apparently eliminated when nicotinic agonist ABS coexists with either 5-CT or CPA**. **(A,B)** (*Left*) Representative paired-pulse AMPAR current traces recorded from the same neuron in the absence (control, black lines) and presence (green lines) of 3 μM ABS plus 10 nM 5-CT **(A)** or 3 μM ABS plus 10 μM CPA **(B)**. Each trace is the average of three consecutive trials. Stimulus artifacts are omitted. (*Right*) Summary plot of the 1st EPSC and the 2nd EPSC in drug relative to that in control and PPR in drug (*n* = 5). The concentrations used are 10 nM, 10 μM, and 3 μM for 5-CT, CPA, and ABS, respectively. The interstimulus interval is 50 ms. Some data of single drugs are from Figure 1. ^**^p < 0.01, compared between the effects with and without the addition of ABS by Student's unpaired *t*-test. See also Figure S1 for the data from individual experiments. **(C)** Plot of the averaged PPR vs. ISI for the control condition (open circles, *n* = 10, data from Figure 3) compared to the simultaneous presence of combined drugs (black circles, *n* = 5). The curve is a fit for the control data of the form: PPR = 0.815[1-exp (*t*/166.7)]. The dashed horizontal lines indicate the levels where PPR or relative EPSC equals 1.

Upon stimulation of the optic tract, excitatory (glutamatergic) AMPA receptor (AMPAR)-mediated postsynaptic currents (EPSCs) were recorded from the thalamocortical relay neuron in the dLGN (Yang et al., 2014). In response to paired stimulation (separated by 50 ms), EPSCs show characteristic short-term depression at this synapse (Figure 1, control). Similar to the previous reports (Chen and Regehr, 2003; Seeburg et al., 2004; Yang et al., 2014), addition of the 5-HT1 receptor agonist 5-carboxytryptamine (5-CT) in a saturating concentration of 10 nM reduces synaptic current by ~50% (Figures 1A, left,B). In contrast, the second EPSC upon the paired stimulation is not significantly altered, resulting in an increased paired-pulse ratio (PPR) or decreased (relieved) short-term synaptic depression by 5-CT. Norepinephrine (NE), another monoamine, shows qualitatively very similar effect (Figures 1A, middle,B). Via activation of presynaptic A1 receptors, adenosine also decreases synaptic currents and short-term synaptic depression at the retinogeniculate synapse in an apparently similar way to monoamines (Yang et al., 2014) (also see Figure 4B below). The decreased synaptic strength and decreased short-term depression is consistent with a decreased probability of neurotransmitter release by monoamines and adenosine. In sharp contrast, either the non-selective cholinergic receptor agonist carbachol or the nicotinic acetylcholine receptor (nAchR) agonist (+)-anabasine (ABS) selectively enhances only the second EPSC amplitude upon paired-pulse stimulation (Figures 1A, right,B). Similar effects can also be obtained with acetylcholine (Ach) in the simultaneous presence of muscarinic antagonist atropine (Figure 1B). Moreover, non-selective muscarinic receptor agonist milameline (up to 30 μM) shows no discernible synaptic effects. These findings suggest that the synaptic modulation is mediated by nicotinic receptors. We therefore examined the synaptic effect with selective nicotinic receptor agonists. Figure 2A shows that an α4β2 nAchR-selective agonist (−)-nicotine ditartrate (ND) shows synaptic effects very much analogous to those of ABS, whereas a highly-selective α7 nAchR agonist PNU 282987 lacks such effects. This finding is consistent with the high-level expression of α4β2 rather than α7 nicotinic receptors in mouse optic nerve axons (Zhang et al., 2004). In addition, ND does not change the first evoked EPSC but selectively increases the subsequent EPSCs in response to either paired or a 50 Hz-train of presynaptic stimuli (Figure 2B). Consistent with the essentially unchanged first EPSC, no significant alteration of the coefficient of variation (CV) of AMPAR EPSC before and after the application of ND is noted (Figure 2B, inset, also see Figure 3F below). The changes in synaptic currents are thus very different with monoaminergic and cholinergic modulation, although PPR is apparently increased in both cases.

### Aminergic modulators and adenosine share very similar effects on retinothalamic transmission

In control, the retinothalamic synapse shows characteristic time-dependent short-term depression, where PPR is smaller with shorter interstimulus intervals (ISI) and increases with ISI lengthening (Figures 3A–C, open circles). The recovery from synaptic depression is accelerated by adenosine as well as monoaminergic and nicotinic modulators (Figures 3A–C). In the presence of either A1 receptor agonist 6-cyclopentyladenosine (CPA, 10 μM) or 5-CT (10 nM), PPR is especially increased with ISIs less than ~500 ms, resulting in an “biphasic” recovery course (Figures 3A,B). It appears that CPA or 5-CT has an effect only on the fast component (i.e., for ISI < 500 ms) of the biphasic paired-pulse kinetics. This finding may indicate a decrease in probability of neurotransmitter release, as it has been reported in other synapses that short-term synaptic facilitation with low probability of release usually recovers faster than short-term depression with high probability of release (Thomson, 2000; Zucker and Regehr, 2002; Fioravante and Regehr, 2011). Consistently, the CV of EPSC is increased by either CPA or 5-CT (Figure 3F). In sharp contrast, nicotinic agonist ABS slightly but consistently increases PPR over a relatively wide range of ISIs (Figure 3C). Also, ABS enhances the second EPSC amplitude with ISIs shorter than 1 s (Figure 3D), but shows no evident effect on basic synaptic strength (i.e., the unchanged first EPSC, Figure 1). Nicotinic activation thus does not seem to alter the initial probability of neurotransmitter release (see also Figures 2, 3F). In this regard, it is interesting that α4β2 nAchR agonist ND fails to significantly increase PPR with lowered concentration (~1 mM) of extracellular Ca^2+^ (Figure 3E). In contrast, 5-CT and CPA modulation of synaptic plasticity (PPR) is not influenced when extracellular Ca^2+^ level is decreased from 2 to 1 mM (Figure 3E). Therefore, in terms of the effects on paired-pulse currents, CV of EPSC, and the time course as well as Ca^2+^ dependence of short-term depression, wake-promoting monoamine is very similar to the presumable somnogen adenosine, but is different from another wake-promoting modulator acetylcholine.

### Cholinergic modulators interact with either aminergic modulator or adenosine to attenuate short-term synaptic plasticity

**Figure 5:**
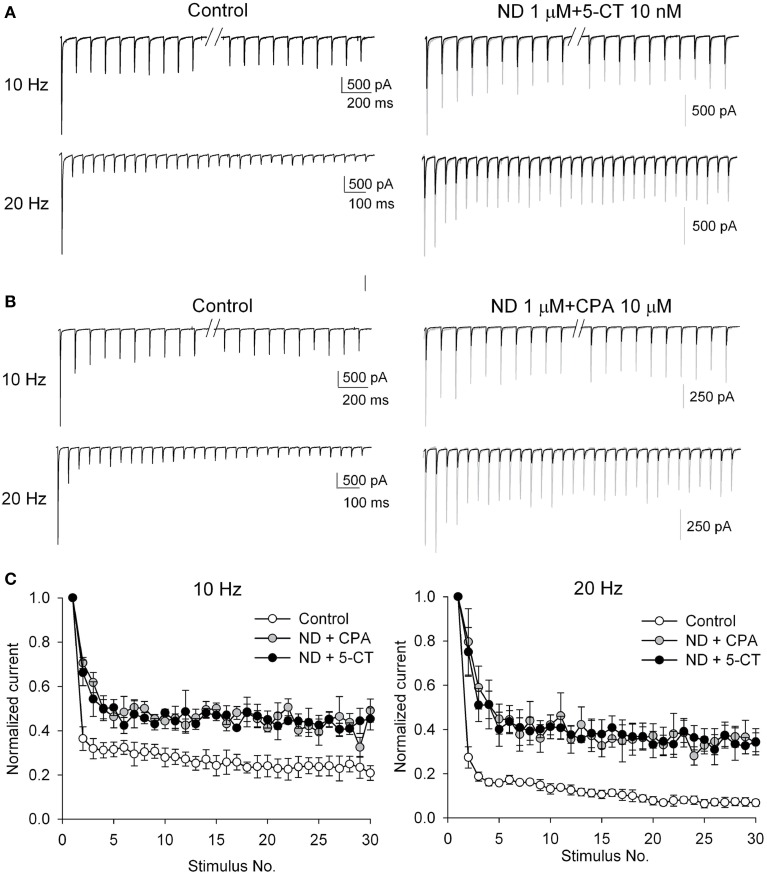
**The short-term synaptic plasticity in response to trains of stimuli is similarly abolished when α4β2 nAchR agonist ND coexists with 5-CT or CPA**. **(A,B)** AMPAR EPSC traces from a representative neuron in control (left panels) or in the presence of 1 μM ND plus 10 nM 5-CT **(A)** or 1 μM ND plus 10 μM CPA **(B)** (right panels, using the same scale bars for left panels) in response to a 10 Hz-(upper panels) or 20 Hz-(lower panels) train of 30-stimuli (black lines). Stimulus artifacts and the responses to the middle 10 stimuli at 10 Hz (i.e., elapsed time = ~1 s) are omitted for clarity. To facilitate comparison, the first EPSC in drug is also scaled to the same amplitude as that in control and shown in gray color. **(C)** Summary of the relative changes in EPSC amplitudes during trains of stimuli at frequencies of 10 Hz (left) and 20 Hz (right). The amplitude of each EPSC is normalized to the first EPSC of the train and is plotted against stimulus number. Data are averaged from four cells for each experiment.

It is intriguing that monoamines and acetylcholine should show distinct individual effects on the retinothalamic synapse, considering that monoamines and acetylcholine are both the dominant neuromodulators in the thalamus during wakefulness (Jouvet, 1969; Cespuglio et al., 1984; Lucas-Meunier et al., 2003; Villablanca, 2004; Saper et al., 2005; Haas and Lin, 2011). We therefore examined the combined effect of serotonin and acetylcholine. Figure 4A shows that application of both nAchR agonist ABS and 5-CT produces more inhibition of EPSC than that by 5-CT alone. On the other hand, the second EPSC upon paired-pulse stimulation is no longer increased with the presence of both ABS and 5-CT, compared to the case with ABS alone. The coexistence of the two modulators thus seems to abolish short-term synaptic plasticity. In the presence of both ABS and 5-CT, the PPR remains close to 1 in all responses to presynaptic stimuli separated by a wide range of ISIs (20–2000 ms, Figure 4C, left), an effect not achievable by any single neuromodulator we examined (even in over-saturating concentrations). On the other hand, bath application of both ABS and CPA results in synergistic effects almost the same as those of application of both ABS and 5-CT (Figures 4B,C). Figures 5A,B further compare EPSCs in response to a 10 Hz- or 20 Hz-train of 30 presynaptic stimuli. In control, the postsynaptic currents are significantly larger if elicited by the earlier than the later pulses, and by 10 Hz- than 20 Hz-train of stimuli, consistent with the “low-pass” transmission through a synapse of short-term depression (Figures 5A,B, left). In the presence of both nicotinic and adenosine receptor agonists or both nicotinic and serotonin receptor agonists, however, the elicited synaptic currents remain similar in amplitude for all pulses at the 10 Hz- or 20 Hz-train of stimuli (Figures 5A,B, right). Also, there are quantitatively similar effects of ND combined with CPA to those of ND combined with 5-CT (Figure 5C). Collectively, short-term synaptic plasticity is much less evident over a wide frequency range (at least 0.5–20 Hz) of presynaptic activity with combined neuromodulators mimicking the scenarios in either the awake or the paradoxical sleep states (see Discussion).

### Adenosine interacts with aminergic modulators to inhibit synaptic strength but reverse the polarity of short-term plasticity

**Figure 6:**
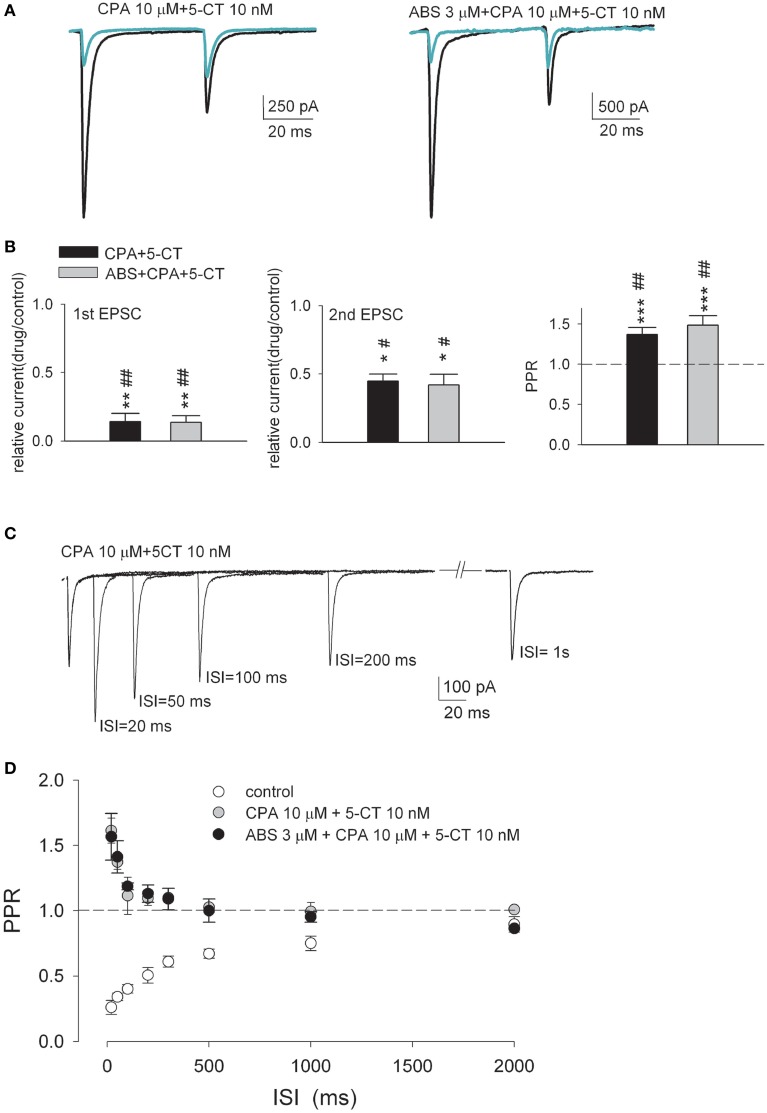
**The short-term depression is transformed into facilitation in the simultaneous presence of both CPA and 5-CT**. **(A)** Representative paired-pulse AMPAR current traces recorded from the same neuron in control (both panels, black lines) and in the presence of 10 μM CPA plus 10 nM 5-CT (left panel, green line) or 10 μM CPA plus 10 nM 5-CT plus 3 μM ABS (right panel, green line). Each trace is the average of three consecutive trials. Stimulus artifacts are omitted for clarity. **(B)** Summary plot of the 1st EPSC (left panel) or the 2nd EPSC (middle panel) in drug relative to that in control or PPR in drug (right panel) (*n* = 5–10). The concentrations used are 10 nM, 10 μM, and 3 μM for 5-CT, CPA, and ABS, respectively. The interstimulus interval is 50 ms. The dashed horizontal line indicates the level where PPR equals 1. ^*^p < 0.05, ^**^p < 0.01, ^***^p < 0.001 when the CPA+5−CT group or the 5−CT+CPA+ABS group are compared to the CPA+ABS group by Student's unpaired *t*-test. #*p* < 0.05, ##*p* < 0.01 when they are compared to the 5-CT+ABS group by Student's unpaired *t*-test. See also Figure S1 for the data from individual experiments. **(C)** Overlay of EPSCs from a representative relay neuron in response to paired pulses of varying ISIs in the presence of 10 μM CPA plus 10 nM 5-CT. Traces are the average of three consecutive trials. Stimulus artifacts are blanked for clarity. **(D)** Plot of the average PPR vs. ISI in the control condition (open circles, *n* = 10, data from Figure 3) and in the presence of 10 μM CPA plus 10 nM 5-CT (gray circles, *n* = 6) or in the presence of 10 μM CPA plus 10 nM 5-CT plus 3 μM ABS (black circles, *n* = 5).

**Figure 7:**
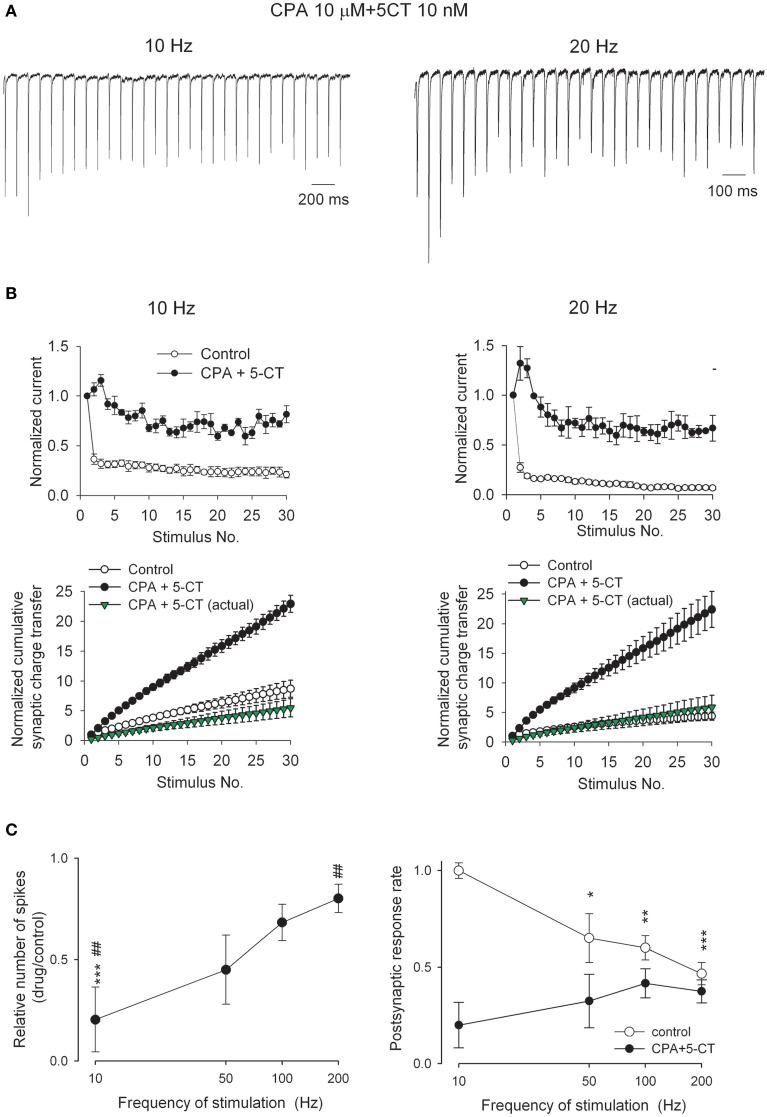
**Short-term synaptic plasticity in response to trains of 30 stimuli at frequencies of 10 and 20 Hz in the presence of 10 μM CPA plus 10 nM 5-CT**. **(A)** AMPAR EPSC traces from a representative neuron in the presence of both CPA and 5-CT. Stimulus artifacts are omitted for clarity. To facilitate comparison, the first EPSCs are scaled to the same amplitude. **(B)** *Upper panel*, Summary of the relative changes in EPSC amplitude during trains of stimuli (*n* = 4). The analyses are the same as those described in Figure 5C. Control data are from Figure 5C. *Lower panel*, The normalized cumulative synaptic charge transfer is calculated from the data in upper panels. Synaptic charge transfer is derived by integration of the postsynaptic current in response to each stimulus normalized to those in response to the first stimulus. In the presence of CPA and 5-CT, synaptic charge transfer in response to each stimulus is also normalized to those in the first pulse in control to obtain an “actual” normalized cumulative synaptic charge transfer (green triangles). **(C)** (*Left*) The number of action potential spikes in the presence of CPA plus 5-CT relative to control is plotted against the stimulation frequency (*n* = 5). The relative number of spikes is significantly reduced in the presence of CPA plus 5-CT (^***^p < 0.001, compared to control by Student's unpaired *t*-test) in response to 10 Hz stimulation. The degree of spike inhibition is significantly less in response to higher when compared to that at lower stimulation frequency in the presence of CPA plus 5-CT (##*p* < 0.01 for 10 vs. 200 Hz). (*Right*) The number of action potential spikes is normalized to the number of presynaptic stimuli and plotted against the stimulation frequency (*n* = 5). ^*^p < 0.05, ^**^p < 0.01, ^***^p < 0.001, compared to the data in response to 10 Hz stimuli by Student's unpaired *t*-test. Bath temperature, 34–36°C.

We have also found that combination of CPA and 5-CT reduces both the first and the second paired-pulse currents much more than in the presence of either one of the two [Figures 6A,B, see also Figures 1B, 4B, and (Yang et al., 2014)]. The inhibition of the second EPSC (~55% in amplitude) is smaller than that of the first (~85% in amplitude, Figure 6B), so that the second EPSC amplitude becomes larger than the first EPSC in the presence of both CPA and 5-CT. Short-term depression is thus transformed into facilitation (i.e., PPR becomes larger than 1, Figure 6B). The much stronger inhibition of the first EPSC and the reversed polarity of short-term plasticity cannot be produced by either CPA or 5-CT alone (Yang et al., 2014), and surprisingly would remain the same with the addition of the third modulator ABS (even in a saturating concentration of 3 μM) (Figures 6A,B). In the presence of both CPA and 5-CT (with or without ABS), a pair of optic nerve stimulation with an ISI of ~20–50 ms evokes a second EPSC ~50% larger than the first EPSC (Figures 6C,D). This short-term synaptic facilitation is gradually decreased with longer ISI, and is almost not discernible with an ISI longer than ~500 ms. The faster recovery kinetics from short-term facilitation than that from short-term depression (e.g., in control) is consistent with what were reported in other synapses with short-term facilitation (Figure 6D, Thomson, 2000; Zucker and Regehr, 2002). Again, the addition of the third modulator ABS does not change the recovery courses. In the presence of both CPA and 5-CT, synaptic facilitation is also demonstrated when the synapse responds to a train of presynaptic activity (Figure 7). Synaptic facilitation is especially evident for the first few presynaptic stimuli, and for 20 Hz rather than 10 Hz stimulation (Figure 7B, upper panels). Despite the very low synaptic strength with the coexistence of CPA and 5-CT, cumulative synaptic charge transfer may still be close to or even exceed control if presynaptic activity is high and long enough (Figure 7B, lower panels).

### Adenosine interacts with aminergic modulators to decrease the fidelity of retinothalamic relay with low- but not high-frequency stimuli

Synaptic strength and short-term plasticity are important factors that influence postsynaptic responses and information processing. We also directly examined how the relay of information encoded by presynaptic activity is altered by the neuromodulators at the retinogeniculate synapse. Upon stimulation of the optic tract at different frequencies, tonic mode of action potential discharges were recorded from a postsynaptic relay neuron that was first depolarized to −55 mV, at which potential CPA or 5-CT do not seem to affect postsynaptic conductance significantly [see Supporting Information Figure S3, (Seeburg et al., 2004)]. Bath application of both CPA and 5-CT markedly reduces action-potential spike numbers of postsynaptic relay neuron in response to a 10 Hz-train (but not so much with ~100–200 Hz-trains) of 30-stimuli (Figure 7C, left). The inhibitory effects are more prominent at lower than higher frequencies of presynaptic stimuli. This frequency-dependent reduction of postsynaptic spikes is consistent with the reversed short-term synaptic plasticity by coexisting 5-CT and CPA. It appears that the transformation from synaptic depression to facilitation compensates for the reduction of synaptic strength, resulting in the maintenance of relay neuron spiking in response to higher presynaptic activity. In other words, this synapse is modulated toward a “high-pass filter.” On the other hand, postsynaptic responsiveness is more dependent on the frequency of presynaptic activity in control than in the presence of both 5-CT and CPA (Figure 7C, right). Coexisting 5-CT and CPA would therefore markedly decrease the fidelity of retinothalamic (and then thalamocortical) coding of sensory information chiefly for the low-frequency external stimuli (see Discussion).

## Discussion

The short-term synaptic plasticity determines how presynaptic spike patterns would influence postsynaptic ones (Zucker and Regehr, 2002). Most synapses have a specific form of short-term plasticity due to their unique presynaptic and/or postsynaptic properties (Regehr and Stevens, 2000; Zucker and Regehr, 2002). Our findings from the retinothalamic synapse support that multiple mechanisms underlying different forms of short-term plasticity could be present in one synapse (von Gersdorff and Borst, 2002) and could be selectively activated in a context-dependent manner by the surrounding neuromodulators. We have chiefly focused on a presynaptic locus of neuromodulatory action in this study because our experimental system minimizes the postsynaptic factors by voltage-clamp of the postsynaptic neuron and by using a Cs^+^-based internal solution (Trussell and Jackson, 1985), and also because the neuromodulators all produce quantitatively similar effects on the strength and plasticity of AMPAR and NMDAR-mediated synaptic currents (Yang et al., 2014 and our unpublished data) (also see Supporting Information Figure S2). Consistent with reduction of presynaptic neurotransmitter release, serotonin or adenosine that decreases synaptic strength would decrease short-term depression, and combination of serotonin and adenosine that robustly decreases synaptic strength would even show short-term facilitation in response to high-frequency stimuli. Moreover, the CV of EPSC is increased by either CPA or 5-CT (Figure 3F). Also, the “biphasic” course of recovery from paired-pulse depression by either CPA or 5-CT supports a decrease in probability of release because a synapse with lower probability of release tends to recover faster (Thomson, 2000; Zucker and Regehr, 2002). On the other hand, activation of presynaptic nicotinic acetylcholine receptors has been shown to increase spontaneous neurotransmitter release or increase axonal excitability in other brain areas (Lambe et al., 2003; Kawai et al., 2007; Lendvai and Vizi, 2008). The selective increase of subsequent release without altering basic synaptic strength at the retinogeniculate synapse would argue against that the observed nicotinic effect is due to alteration of axonal excitability. Instead, nicotinic agonists may accelerate replenishment of a depleted vesicle pool or enhance accumulation of (or increase sensitivity to) presynaptic Ca^2+^ to increase the response to the second stimulus, and thus their effect on PPR would be less prominent with lower concentrations of extracellular Ca^2+^ (Figure 3E) (Katz and Miledi, 1968; Zucker and Regehr, 2002; Catterall and Few, 2008; Mochida et al., 2008; Neher and Sakaba, 2008; Nakajima et al., 2009; Fioravante and Regehr, 2011). Consistently, it has been shown that nicotine might elevate calcium influx into optic nerve axons (Zhang et al., 2004). In contrast, neither CPA nor 5-CT nor ND alters the postsynaptic membrane potential at around −55 mV significantly (Supporting Information Figure S3) (Seeburg et al., 2004). On the other hand, all modulators examined tend to increase PPR without slowing down the decay of AMPAR currents. These findings suggest a presynaptic site of action of the neuromodulators characterized in this study.

Monoamines and acetylcholine are the dominant neuromodulators in the thalamus with sanity of mind or during wakefulness (Jouvet, 1969; Cespuglio et al., 1984; Lucas-Meunier et al., 2003; Villablanca, 2004; Saper et al., 2005; Haas and Lin, 2011), but show distinct individual effects on the retinothalamic synapse (Figures 1–3). While monoamine decreases the release of neurotransmitters, acetylcholine increases synaptic transmission as what were reported in many other brain areas (Vidal and Changeux, 1993; McGehee et al., 1995; Gray et al., 1996; Wonnacott, 1997; Miyazaki et al., 1998; Forray et al., 1999; Delaney et al., 2007; Fink and Göthert, 2007). The distinct synaptic effects suggest different physiological roles played by the two dominant neuromodulators in the thalamus during wakefulness. It seems plausible that monoamine ensures more precise relay of presynaptic activities to the postsynaptic site by limiting the size of transmission and decreasing synaptic depression, whereas acetylcholine tends to facilitate transmission more generally. When monoamines and acetylcholine coexist, however, we found that monoamines apparently trim down the generally promoted neurotransmission by acetylcholine in a non-additive fashion at the retinogeniculate synapse. The combination leads to further decrease of synaptic strength as well as less short-term depression, resulting in largely abolished short-term synaptic plasticity. Such neuromodulatory effects may especially reduce the transmission of low-frequency noise (low-amplitude stimuli) and therefore allow more precise and undisturbed coding of the presynaptic information.

The concentration of adenosine significantly increases in many brain areas after prolonged wakefulness (e.g., just before the onset of natural sleep) (Porkka-Heiskanen et al., 1997, 2000, 2002; Dobolyi et al., 2000; Strecker et al., 2000; Monti and Jantos, 2008; Elmenhorst et al., 2009; Lin et al., 2011). In terms of individual effect, adenosine and serotonin are very much alike in almost all aspects examined in this study (Figures 1, 3, 4) (Yang et al., 2014). Intriguingly, the combination of adenosine and monoamines leads to synergistic synaptic effects (Yang et al., 2014) (also see Figure 6). EPSCs are largely inhibited, and the polarity of short-term plasticity is reversed. The combined actions of the two modulators are consistent with a much lowered probability of glutamate release, which would impair synaptic charge transfer but favor a facilitation form of short-term plasticity (Katz and Miledi, 1968; Thomson, 2000; Zucker and Regehr, 2002). On the one hand, the thalamic synaptic transmission would be markedly downscaled (Tononi and Cirelli, 2003, 2006), leading to the very much reduced transmission of sensory information through the thalamus appropriate for the slow-wave sleep state, when adenosine and monoamines very likely coexist. On the other hand, short-term synaptic facilitation allows transmission of presynaptic activity that encodes sensory information in high-enough frequency and long-enough duration (Figures 6, 7), and even awakening may therefore ensue. Because the markedly decreased synaptic strength and the reversed polarity of short-term plasticity occur only when monoamines coexist with adenosine, the existence of wake-promoting monoamines in the NREM sleep state (when the aminergic tone is lowered but by no means absent) could actually be important for appropriate information processing in the thalamus for that state. This may well explain the puzzling apparent contradictory roles of serotonin in sleep and wakefulness (Jouvet, 1999). Moreover, moderate fluctuations in acetylcholine level are probably allowed in this particular state, given that the presence of acetylcholine does not significantly alter the combined synaptic effect of serotonin and adenosine (Figure 6). On the other hand, we found that under the influence of coexisting adenosine and acetylcholine (but not monoamines, which are absent as in the REM state), the apparent properties of thalamic synaptic transmission are very similar to that with serotoninergic and cholinergic coactivation during wakefulness (Figures 4, 5). This is reminiscent of the similar electroencephalographic (EEG) patterns and thalamocortical activity patterns found in the waking and in the REM sleep states (McCormick and Bal, 1997; Poulet et al., 2012). The presence of acetylcholine and adenosine, and equally or even more importantly, the absence of monoamines, are therefore the prerequisites for the occurrence of REM sleep from the view point of synaptic transmission in the thalamus. Given a very similar effect of synaptic modulation between adenosine plus acetylcholine and serotonin plus acetylcholine, adenosine may be envisaged as a replacement of monoamine, which is not allowed to be present in the REM sleep state, to have a higher level of thalamic synaptic transmission and activation of many parts of thalamocortical networks in REM sleep comparable to the wakefulness state.

We conclude that thalamocortical neuronal responses to network influences can be finely tuned by both presynaptic electrical activity and neuromodulators from distant and local sources. The actions of these key modulators have been mostly ascribed to the modification of thalamic neuronal membrane conductance previously (McCormick, 1992). We would propose a novel perspective that the concerted actions of different neuromodulators on synaptic transmission could also sum up to play a fundamental role in the control of oscillatory patterns and appropriate information processing in the thalamocortical networks.

### Conflict of interest statement

The authors declare that the research was conducted in the absence of any commercial or financial relationships that could be construed as a potential conflict of interest.

## Abbreviations

ABS: (+)-anabasine
Ach: acetylcholine
AMPAR: the AMPA receptor
REM: rapid-eye movement
2-CA: 2-chloro-N6-cyclopentyladenosine
CPA: N6-cyclopentyladenosine
5-CT: 5-carboxytryptamine
CV: coefficient of variation
dLGN: dorsal lateral geniculate nucleus
EEG: electroencephalic graph
EPSC: excitatory postsynaptic current
ISI: interstimulus interval
nAchR: nicotinic acetylcholine receptor
ND: (−)-nicotine ditartrate
NE: norepinephrine
NREM: non-REM
PPR: paired-pulse ratio.
