# Supplementary material for: Non-additive modulation of synaptic transmission by serotonin, adenosine, and cholinergic modulators in the sensory thalamus
Source: Front Cell Neurosci. 2015 Mar 16;9:60. doi: 10.3389/fncel.2015.00060 (PMC4360759; doi:10.3389/fncel.2015.00060)
Supplement: Supplementary file 1 [file DataSheet1.PDF]

Supporting Information

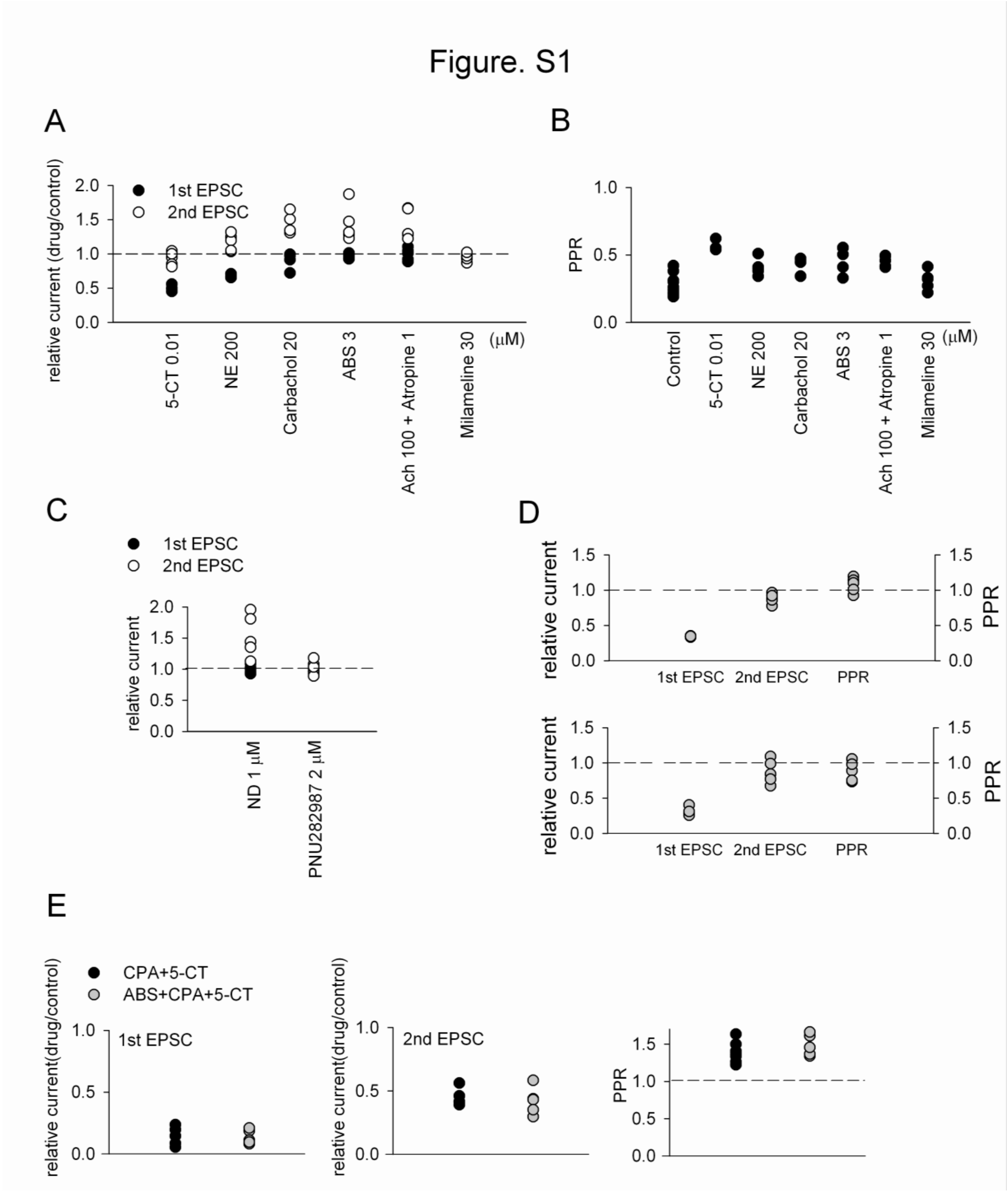

Figure S1: The data from individual experiments that constitute the bar graphs in Figs. 1B (left panel), 1B (right panel), 2A, 4A (right panel, the data of ABS+5-CT), 4B (right panel, the data of ABS+CPA), and 6B are plotted in parts A, B, C, D (upper panel), D (lower panel), and E, respectively, to demonstrate the congruency.

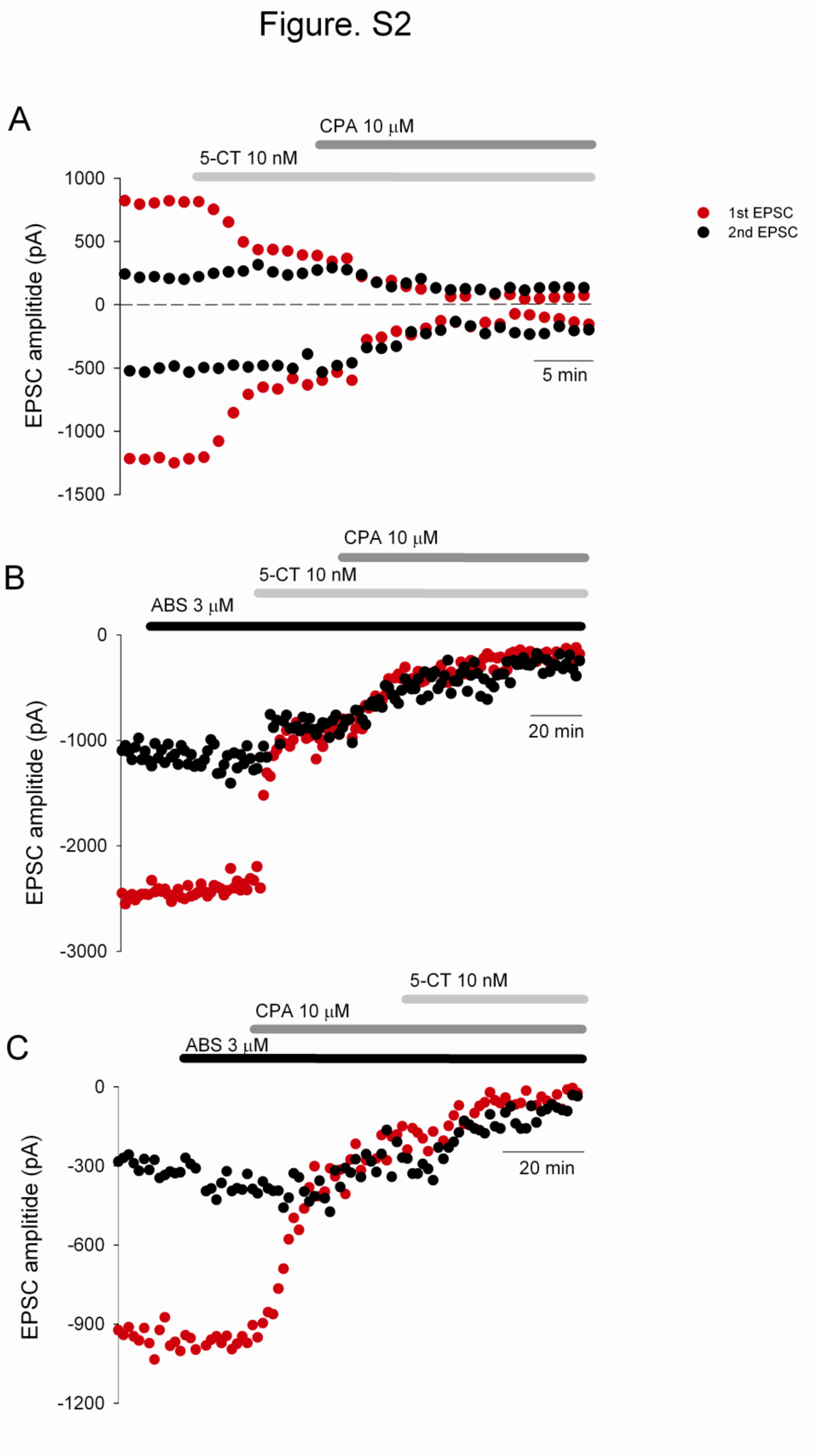

Figure S2: The time course of retinogeniculate synaptic modulation by bath application of different modulators. (A) The peak amplitude of the first (red dots) and the second (black dots) EPSCs

elicited by paired pulse stimulation (interstimulus interval: 50 ms) through the AMPA (negative currents) and the NMDA (positive currents) receptors are plotted over time before and during the sequential application of 10 nM 5-CT and then 10 μM CPA plus 10 nM 5-CT. (B) Similar plot of AMPAR EPSCs except that 3 μM ABS is applied first, followed by 3 μM ABS plus 10 nM 5-CT, and then 3 μM ABS plus 10 nM 5-CT plus 10 μM CPA. (C) Similar plot of AMPAR EPSCs except that 3 μM ABS is applied first, followed by 3 μM ABS plus 10 μM CPA, and then 3 μM ABS plus 10 nM 5-CT plus 10 μM CPA.

Figure S3

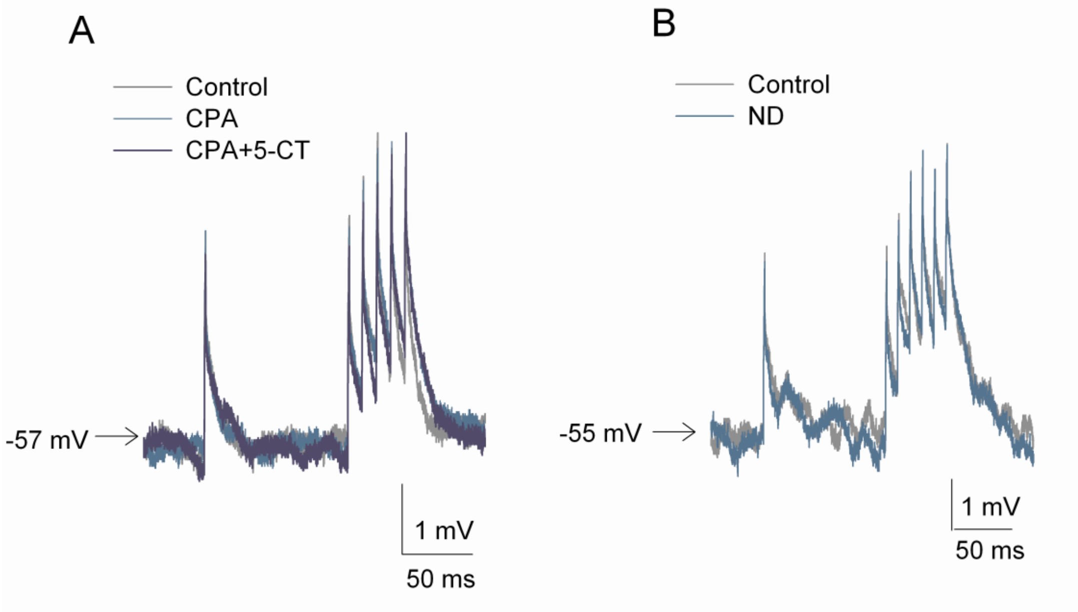

Figure S3: CPA, 5-CT, or ND does not significantly alter postsynaptic membrane potential in response to current injections. At membrane potential of ~-55 mV, a representative dLGN neuron is injected by a short pulse current followed by a 100 Hz train of five or six pulses in the absence (grey lines) and the presence of 10 μM CPA (part A, cyan line), 10 nM 5-CT plus 10 μM CPA (part A, blue line), or 1 μM ND (part B, cyan line). See Materials and Methods in the text for the current-clamp recording methods.
